# Construction of Hexagonal Prism-like Defective BiOCL Hierarchitecture for Photocatalytic Degradation of Tetracycline Hydrochloride

**DOI:** 10.3390/nano12152700

**Published:** 2022-08-05

**Authors:** Lijun Hu, Zhichao Ding, Fei Yan, Kuan Li, Li Feng, Hongqing Wang

**Affiliations:** 1 School of Chemistry and Chemical Engineering, University of South China, Hengyang 421001, China; 2 Hunan Key Laboratory for the Design and Application of Actinide Complexes, University of South China, Hengyang 421001, China; 3 School of Civil and Transportation Engineering, Guangdong University of Technology, Guangzhou 510006, China

**Keywords:** BiOCl, photocatalytic, lattice defects, hierarchical structure, degradation, ambient pressure

## Abstract

Oxygen vacancy manipulation and hierarchical morphology construction in oxygen-containing semiconductors have been demonstrated to be effective strategies for developing high efficiency photocatalysts. In most studies of bismuth-based photocatalysts, hierarchical morphology and crystal defects are achieved separately, so the catalysts are not able to benefit from both features. Herein, using boiling ethylene glycol as the treatment solution, we developed an etching-recrystallization method for the fabrication of 3D hierarchical defective BiOCl at ambient pressure. The target hierarchical 3D-BiOCl is composed of self-assembled BiOCl nanosheets, which exhibit a hexagonal prism-like morphology on a micron scale, while simultaneously containing numerous oxygen vacancies within the crystal structure. Consequently, the target catalyst was endowed with a higher specific surface area, greater light harvesting capability, as well as more efficient separation and transfer of photo-excited charges than pristine BiOCl. As a result, 3D-BiOCl presented an impressive photocatalytic activity for the degradation of tetracycline hydrochloride in both visible light and natural white light emitting diode (LED) irradiation. Moreover, an extraordinary recycling property was demonstrated for the target photocatalyst thanks to its hierarchical structure. This study outlines a simple and energy-efficient approach for producing high-performance hierarchically defective BiOCl, which may also open up new possibilities for the morphological and crystal structural defect regulation of other Bi-based photocatalysts.

## 1. Introduction

Advanced oxidation processes of semiconductor photocatalysis have been deemed a viable strategy for removing toxic and poisonous compounds owing to their low energy consumption and environmental protection features [1,2,3]. Researchers have exploited a variety of efficient photocatalysts in the past to cover the demands, including TiO_2_, ZnO, CeO_2_, CdS, and C_3_N_4_ [4,5,6,7,8,9]. Among these, bismuth oxychloride (BiOCl) has emerged as one of the most promising photocatalysts in recent years owing to its unique layered structure [Cl-O-Bi-O-Cl] and elastic optoelectronic properties [10,11,12]. It has been thoroughly demonstrated over the past years that the self-constructed internal electric field along the direction of [001] is capable of greatly facilitating electron–hole pair separation, allowing the possibility for BiOCl to achieve high quantum efficiency levels. Moreover, the low bond energy and long bond length of the Bi-O bonds in BiOCl enable the development of deficient photocatalysts more easily than traditional catalysts. Up to now, considerable development has been made in the field of BiOCl photocatalysts, which have been successfully applied in photocatalytic hydrogen and oxygen production, pollutant degradation, nitrogen fixation, carbon dioxide reduction, selective organic transformation, and other applications [9,13,14,15]. Yet, despite this, the broad band gap energy of BiOCl (3.27 eV) and the high rate of charge recombination under solar light limit its efficiency as a photocatalyst [16,17].

To meet the aforementioned challenge, numerous strategies have been proposed for extending the light absorption range and suppressing carrier recombination of BiOCl photocatalysts, such as doping, loading a noble metal, modifying the morphology, or combining with other photocatalysts [17,18,19,20,21]. Modifications involving morphological engineering have been shown to be effective for enhancing photocatalytic activity. In a study by Huang et al., for example, in situ topotactic transformation of BiVO_4_ was used to obtain ultrathin BiOCl nanosheets, which increased the specific surface area of specimens and ciprofloxacin degradation efficiency compared with the bulk BiOCl [22]. Using a wet chemical approach, Zhang and co-workers constructed BiOCl nanosheets with periodic nano-channels, which presented a longer lifetime of excitons than the control sample [23]. In consequence, the target photocatalyst outperformed the control specimens in photo-oxidation of benzylamine coupling by a factor of 30. As well as controlling the morphology, generating oxygen vacancies (OVs) in the crystal structure is an efficient means for improving the photocatalysis efficiency of specimens. On one side, OVs can create defective energy levels in the forbidden band, thus extending the photo-absorption range [24,25,26,27]. The OVs on the surface of the photocatalyst, on the other hand, can serve as reactive sites for the absorption and activation of reactive species [9,28]. However, in most studies of bismuth-based photocatalysts, hierarchical morphology and crystal defects are achieved separately, so the catalysts are not able to benefit from both features. Therefore, it is particularly appealing to endow a specific hierarchical shape and lattice defect simultaneously in the BiOCl photocatalyst to expose more reaction sites and enhance charge transfer efficiency during the photocatalytic process.

In this work, we develop a simple etching-recrystallization process at ambient pressure for obtaining a three-dimensional hierarchical defect BiOCl photocatalyst (abbreviated as 3D-BiOCl). The results of the study indicate that 3D-BiOCl constructed in boiling ethylene glycol (EG) exhibits oxygen vacancies in abundance and a hierarchical hexagonal morphology at the micron level. Such features enhance its specific surface area, solar light absorption, as well as the separation and transfer of photo-excited charges. As a consequence, the target 3D-BiOCl photocatalyst exhibits significantly greater tetracycline hydrochloride degradation efficiency under visible and natural white LED light than pristine BiOCl. Furthermore, owing to the hierarchical structure of the target photocatalyst, it demonstrates excellent recycling potential as well. Our findings, we believe, may also provide insight into the morphological and crystal structural defects regulation of other Bi-based photocatalysts.

## 2. Experimental

### 2.1. Materials

Bismuth trioxide (Bi_2_O_3_, AR), ethanol (C_2_H_6_O, 99.7% purity), ethylene glycol (EG, AR, 99% purity), and tetracycline hydrochloride (C_22_H_24_N_2_O_8_·HCl, 96% purity) were purchased from Aladdin Reagents Co., Ltd. (Shanghai, China), and all the chemical reagents were used as received without further treatment or purification.

### 2.2. Preparation of Pristine BiOCl Nanosheets

The BiOCl nanosheets, according to previous work, were prepared using a modified hydrothermal method. The typical process for synthesizing BiOCl nanosheets starts with 0.968 g (2 mmol) bismuth nitrate pentahydrate (Bi(NO_3_)_3_·5H_2_O) being poured into 50 mL ultrapure water and vigorously stirred [29]. Afterward, 10 mL of saturated sodium chloride (NaCl) was added to the above solution to form a white suspension. After the mixture was processed, it was placed in a Teflon-lined stainless steel autoclave and maintained at 160 °C for three hours. As soon as the reactor cools to ambient temperature, the white product BiOCl is centrifuged and rinsed with ultrapure water and ethanol five times before being dried at 100 °C for 12 h.

### 2.3. Preparation of 3D-BiOCl Photocatalyst

In this study, 3D-BiOCl was received by etching pristine BiOCl and re-crystallizing it at atmospheric pressure. In detail, 30 mL of ethylene glycol was first added to a 100 mL flat bottom beaker and boil on a hotplate. Following this, 0.5 g of pristine BiOCl powder was added directly to the boiling solution and etched for 30 min to construct the target photocatalyst. Finally, the 3D-BiOCl photocatalyst was collected following centrifugation, rinsed with ultra-pure water and ethanol four times, and then dried for 12 h at 100 °C in a vacuum oven.

### 2.4. Characterization

X-ray diffraction (XRD) patterns were recorded using a PANalytical Empyrean diffractometer (PANalytical, Almelo, the Netherlands) with Cu Kα radiation (λ = 1.5406 Å). Scanning electron microscopy (SEM) and energy dispersive spectroscopy (EDS) images were recorded by a field emission scanning electron microscope (JSM-7800F, JEOL Ltd., Tokyo, Japan). The steady-state photoluminescence (PL) spectra were collected using a fluorescence spectrophotometer (Cary Eclipse, Agilent, Santa Clara, CA, USA) with an excitation wavelength of 300 nm. Raman spectra were obtained using a confocal Raman Microscope LabRAM HR Evolution (HORIBA, London, UK) equipped with a laser emitting at 514 nm. X-ray photoelectron spectroscopy (XPS) data were acquired in the Thermo Scientific K-Alpha XPS spectrometer (Thermo Fisher Scientific Inc., Waltham, MA, USA). A CHI660E electrochemical workstation with a three-electrode cell was used to measure the electrochemical impedance spectroscopy (EIS) and photocurrent. The optical absorption and nitrogen adsorption–desorption isotherms of photocatalysts were collected on UV-3600 UV/Vis diffuse reflectance spectrophotometer (Shimadzu, Tokyo, Japan) and Micrometritics ASAP 2460 Surface Area and Porosity Analyzer (Micromeritics ASAP 2460, Norcross, GA, USA), respectively. On a Bruker EMX Plus spectrometer (Karlsruhe, Germany), electron paramagnetic resonance (EPR) spectra were collected.

### 2.5. Photocatalytic Measurements

The photocatalytic activities of BiOCl and 3D-BiOCl were evaluated by degradation of tetracycline hydrochloride (TC-HCl) under visible light and LED light. For LED light and visible light, a 55 W 4000K LED light (YMZB-GDZN, Zhongshan, China YAME) and a 300 W xenon lamp with a 420 nm filter (HSX-F300, Beijing NBet), respectively, were employed. In a typical photocatalytic process, 100 mg of photocatalyst is dispersed into 100 mL of 30 mg/L TC-HCl solution in a 250 mL cylindrical glass reactor, followed by magnetic stirring for 60 min under darkness to establish an adsorption–desorption equilibrium. After that, the reaction cell was transferred to a distance of about 30 cm from the light source to perform photocatalytic degradation of TC-HCl. During the photocatalytic process, 2 mL dispersion was sucked out to measure the concentration of TC-HCl solution by UV/Vis spectrophotometer every 10 min (λ= 358 nm). Figure S1 depicts the UV/Vis absorbance spectrum of TC-HCl, which exhibits an obvious characterized absorption peak at 358 nm.

## 3. Results and Discussion

### 3.1. Morphology and Structure of Photocatalysts

**Figure 1:**
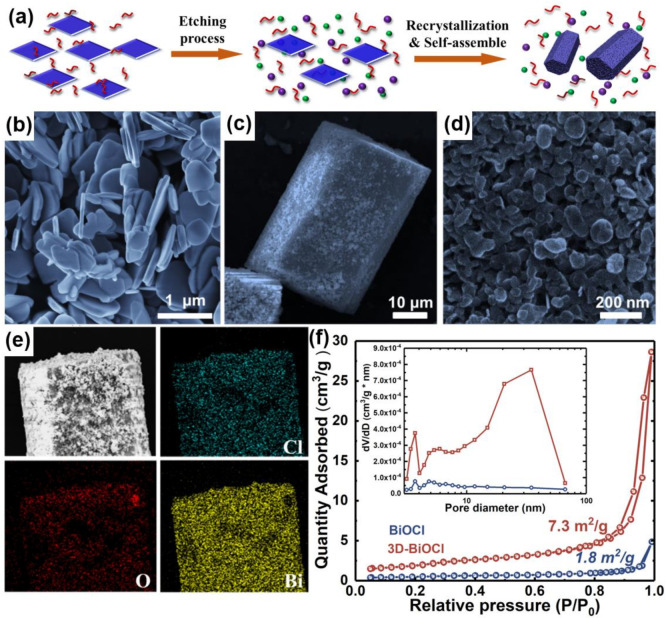
(**a**) Schematic illustration of the 3D-BiOCl fabrication process; SEM pictures of 3D-BiOCl (**b**–**d**); (**e**) Cl, O, and Bi element mappings of 3D-BiOCl specimen; and (**f**) N_2_ adsorption–desorption isotherms and pore size distributions (inset) of BiOCl and 3D-BiOCl.

In this study, a 3D-BiOCl photocatalyst was prepared by etching BiOCl nanosheets in boiling EG followed by recrystallization of the etching products, as indicated schematically in Figure 1. The morphologies and component distribution of the BiOCl and 3D-BiOCl were first visualized by SEM with EDS. According to Figure S2, the bulk BiOCl product obtained by hydrothermal preparation was composed of numerous nanoplates with an average size of around 800 nm. Compared with the typical lamellar morphology of pristine BiOCl, the as-obtained 3D BiOCl sample exhibited a striking hexagonal prism structure, with a length of up to tens of micrometers, as exhibited in Figure 1a,b and Figure S3. It is interesting to note that a high-magnification SEM image in Figure 1c clearly demonstrates that the 3D-BiOCl hexagonal prism was assembled by small nanoplates with a width within a few tens of nanometers. Such a hierarchical structure can effectively prevent the stacking of nanosheets, resulting in a high surface area and excellent recyclability for photocatalysts [30,31].

To further confirm the 3D hierarchical structure of re-crystallized specimens, nitrogen adsorption–desorption isotherms were carefully measured at 77 K. As depicted in Figure 1f, the Brunauer Emmett Teller (BET) surface area of the pristine BiOCl was only 1.8 m^2^∙g^−1^, which is in line with previous work. Fascinatingly, an even higher specific surface area (7.3 m^2^∙g^−1^) and a larger mesoporous volume were characterized in 3D-BiOCl, which indicates that the hierarchical structure of microns 3D-BiOCl is not limited to the surface, but is present throughout the whole structure. Furthermore, the hysteresis loop in the range of 0.8–1.0 P/P_0_ as well as the distribution of pore sizes is also indicative of a mesoporous structure characteristic of 3D-BiOCl. The distributions of the elements in 3D-BiOCl were mapped utilizing the EDS mapping technology developed by SEM, as shown in Figure 1e. As can be seen, Bi, Cl, and O elements are distributed homogeneously throughout the target sample, and no impurities have been detected. Based on the results of the foregoing studies, it can be concluded that, by re-crystallizing pure BiOCl in boiling EG, a hexagonal prism-like 3D hierarchical BiOCl was formed.

**Figure 2:**
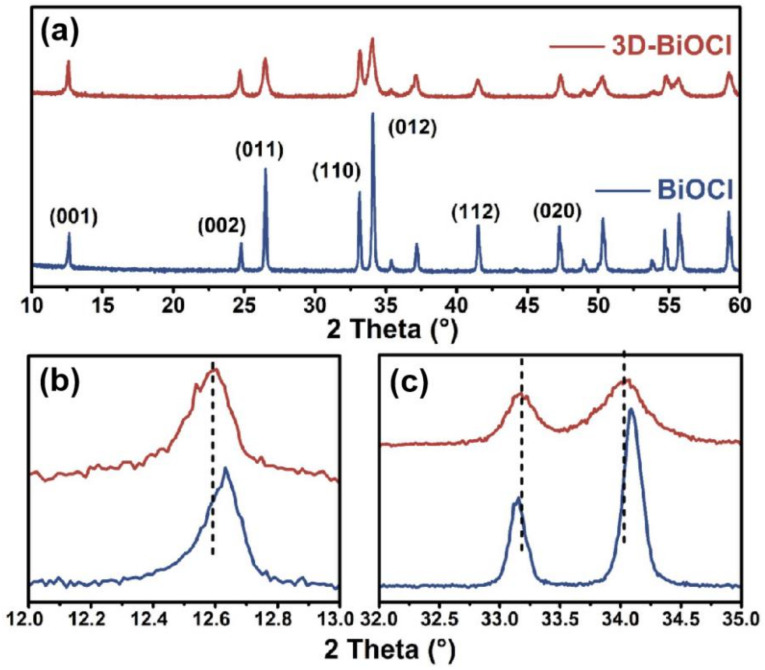
(**a**) XRD diffraction patterns of BiOCl (blue) and 3D-BiOCl (red); (**b**,**c**) zoomed-in images of XRD spectra in the range of 12–13°and 32–35°, respectively.

Upon recognizing the significant morphological difference between BiOCl and 3D-BiOCl, X-ray diffraction (XRD) patterns with a 2θ range of 10–60° were collected to explore the influence of the recrystallization process on the phase composition and crystallographic structure. Figure 2a shows that all of the diffraction peaks are completely indexed to a tetragonal structure typical for BiOCl (JCPDS No.85–0861), with no traces of impurities, thereby indicating both specimens are composed entirely of pure BiOCl [32,33]. In contrast to the identical phase composition, 3D-BiOCl displayed a widening of the diffraction peaks over pure BiOCl, indicating that the re-crystallized sample had a smaller crystal size, which is consistent with observations of SEM. Moreover, differences are more exposed when the details of diffraction peaks are analyzed, as presented in Figure 2b,c. Compared with pristine BiOCl, a distinct shift in diffraction peaks occurs in 3D BiOCl, in which the (001) and (012) peaks shift to a smaller diffraction angle, whereas the (110) peak shifts to a larger diffraction angle. The shift of (001), (012), and (110) peaks is about 0.06°, 0.03°, and 0.07°, respectively, in excess of the instrument bias of about 0.02°, suggesting an anisotropic distortion in the 3D-BiOCl crystal lattice, which is typically caused by lattice defects, such as substitutions and vacancies in the crystal [34,35,36].

**Figure 3:**
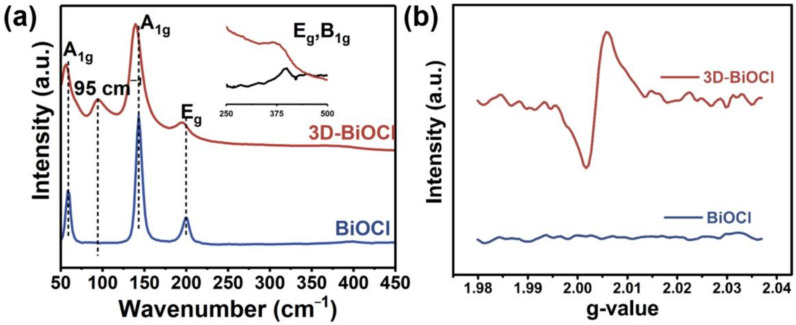
(**a**) Raman and (**b**) EPR spectra of BiOCl and 3D-BiOCl specimens.

Re-crystallization of BiOCl resulted in micro-structural changes and shifted diffraction peaks, which were further investigated using Raman and electron paramagnetic resonance (EPR). Figure 3a provides the representative Raman spectroscopic results of as-prepared samples in the range of 50–450 cm^−1^. Four characteristic vibration bands were detected in virgin BiOCl at 397.8, 199.4, 143.3, and 58.4 cm^−1^, which are consistent with the majority of prior studies on BiOCl. Specifically, the peak at 397.8 cm^−1^ is corresponds to the B_1g_ and E_g_ of the motorial oxygen atoms; the peak at 199.2 cm^−1^ is allocated to the A_1g_ internal stretching modes of Bi-Cl; and the peaks at 58.4 cm^−1^ and 143.3 cm^−1^ pertain to A_1g_ external and internal stretching modes of Bi-Cl, respectively [35,37]. When comparing 3D-BiOCl to BiOCl, three major differences in the spectrum were identified, including a blue-shift in the vibration peak, broadening of peaks, and the creation of a new peak at 95 cm^−1^. The newly discovered vibration band at 95 cm^−1^ is produced from the E_g_ and A_1g_ vibration modes of Bi metal, revealing that 3D-BiOCl contains crystal defects as well as trace amounts of reduced Bi [38]. Additionally, the vibration band broadening and position shifting may be related to the novel shape and oxygen vacancy of the 3D-BiOCl sample [39,40]. A similar phenomenon was also observed in ultra-thin and defective BiOCl [41]. As an effective approach for probing electrons trapped in oxygen vacancies, EPR was adopted to obtain more intuitive evidence for oxygen vacancies in 3D-BiOCl. When compared with pristine BiOCl, the EPR results of 3D-BiOCl demonstrate a distinct signal at g = 2.003 attributed to the oxygen vacancy trapped electrons, as seen in Figure 3b. This finding further shows that, during the re-crystallization process, oxygen vacancies were introduced into the crystal structure of BiOCl.

### 3.2. Photocatalytic Activity and Stability of Photocatalysts under Visible Light

**Figure 4:**
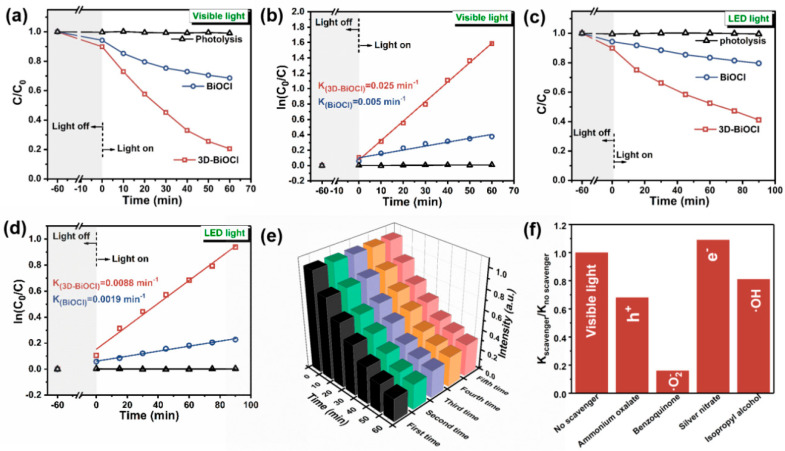
Photocatalytic tetracycline hydrochloride (TC-HCl) degradation under visible light (**a**), natural white LED (**c**), and (**b**,**d**) corresponding pseudo-first-order kinetic fitting curves; (**e**) cycle studies on TC-HCl degradation by 3D-BiOCl under visible light; (**f**) photocatalytic removal of TC-HCl in the presence of different scavengers by 3D-BiOCl under visible light.

To disclose the role of the hierarchical structure and oxygen vacancy of 3D BiOCl in affecting photocatalysis, tetracycline hydrochloride (TC-HCl) was selected as a target contaminant to evaluate the photocatalytic activity and stability of as-obtained samples. Figure 4a depicts a representative temporal evolution curve of the relative concentration of TC-HCl solution with or without a photocatalyst. Before being exposed to visible light, the solution was vigorously stirred in the dark for 60 min to achieve adsorption–desorption equilibrium, and the quantity of TC-HCl uptake by the BiOCl samples was determined using the equation q(TC−HCl)=(V*dC)/m(BiOCl). In agreement with the surface area of specimens, the obtained adsorption quantity of 3D-BiOCl is 3.3 mg·g^−1^, which is larger than the 1.8 mg·g^−1^ of BiOCl. Upon exposure to visible light for one hour, the self-photo-degradation of TC-HCl is almost negligible, indicating its excellent photo-stability. In contrast, using BiOCl and 3D-BiOCl as photocatalysts, an apparent degradation of tetracycline was observed. In contrast, when 3D-BiOCl or BiOCl photocatalyst was added to the reaction, tetracycline was degraded. Using 3D-BiOCl as the photocatalyst, 80 percent of tetracycline was degraded within one hour of exposure to visible light in comparison with merely 31 percent for BiOCl in the same execution environment, suggesting superior photocatalytic degradation ability for 3D-BiOCl. Moreover, the TC-HCl degradation results were subjected to kinetic fitting in order to quantitatively compare the catalytic rates of the samples. As shown in Figure 4b, the degradation curves of both specimens could be well described by the pseudo-first-order kinetics model, and the degradation rates of BiOCl and 3D-BiOCl extracted from the linear fitting between Ln (C/C_0_) and time were 0.005 and 0.025 min^−1^, respectively. It is worth noting that 3D-BiOCl exhibits higher photocatalytic degradation activity with visible light than most other photocatalysts, especially for Bi-based photocatalysts (Table 1). Further, BiOCl pearlescent powder is a potential home decorative material thanks to its non-toxic and environmentally friendly properties. Therefore, it is critical that this material exhibits photocatalytic activity in an interior setting. For this purpose, a natural white LED with 4000 K bulbs was used as the light source to assess the TC-HCl photo-degradation capacities of samples in a home environment. According to Figure 4c, after 90 min of LED illumination, 21 and 59 percent of tetracycline were degraded using BiOCl and 3D-BiOCl as photocatalysts, with corresponding degradation rates of 0.0019 and 0.0088 min^−1^, respectively. Thus, it can be concluded that 3D-BiOCl fabricated by re-crystallization of the original BiOCl is more photo-catalytically active under both visible and natural white (LED) light.

Stability, in addition to photocatalytic activity, is a critical factor to consider when evaluating a photocatalyst. Recycling studies for TC-HCl photo-degradation under visible light irradiation were carried out to evaluate the photo-stability of 3D-BiOCl, as shown in Figure 4e. As can be observed, there was no discernible decline in photocatalytic activity between each cycle, indicating that the 3D-BiOCl photocatalyst was excellently stable throughout photocatalysis. The unique hierarchical structure of 3D-BiOCl, which results in distinguished settling properties, may be responsible for its excellent stability and recyclability. For further investigation of the active species participating in the photocatalytic process, ammonium oxalate (AO), benzoguinone (BQ), silver nitrate (AgNO_3_), and isopropyl alcohol (IPA) were utilized as holes (h^+^), superoxide radical anion (O_2_^−^), electrons (e^−^), and hydroxyl radical (•OH) radical scavengers, respectively. With the addition of e^−^ and •OH scavengers, the degradation activity of TC-HCl by 3D-BiOCl does not appear to differ significantly, suggesting that e^−^ and •OH are not the primary radicals in the catalytic process, as shown in Figure 4f. In contrast, when scavengers of •O_2_^−^ and h^+^ were added to the reaction system, TC-HCl degradation behavior was severely suppressed, with only 16 and 68 degradation rate percentages of the control condition, respectively, indicating that radicals of •O_2_^−^ and h^+^, particularly •O_2_^−^, are essential for visible-light-driven TC-HCl degradation.

**Figure 5:**
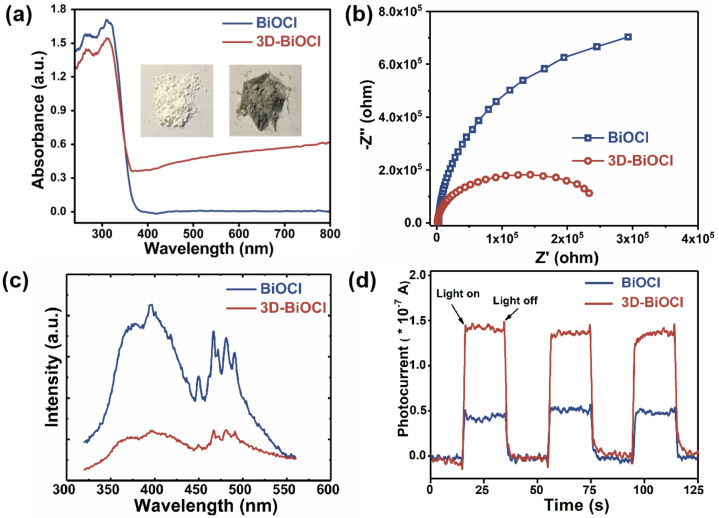
(**a**) UV/Vis absorption, (**b**) electrochemical impedance spectra, (**c**) photoluminescence, and (**d**) transient photocurrent spectra of as-prepared BiOCl photocatalysts.

To provide insight into the underlying reasons for the superior photocatalytic activity of the target specimens, UV/Vis absorption, photoluminescence (PL), and photo-electro-chemical characteristics of BiOCl and 3D-BiOCl were investigated. Generally, lattice defects in semiconductor material would result in changes in the energy band structure, so as to alter the light absorption capabilities of material [15,51,52]. To this end, UV/Vis diffuse reflectance spectra were first performed to investigate the optical absorption properties of BiOCl and 3D-BiOCl. As demonstrated in Figure 5a, 3D-BiOCl exhibits a slightly larger optical absorption edge of 374 nm than BiOCl, which is 365 nm. Moreover, the bandgap energies of the BiOCl samples were further calculated by plotting (αhν)^1/2^ versus photon energy (hν), as shown in Figure S4. According to the intercepts of the tangent to the x-axis, the Eg values of BiOCl and 3D-BiOCl were roughly 3.25 and 3.11 eV, respectively [53]. Nevertheless, 3D-BiOCl is still incapable of absorbing energy from visible light, implying that the tiny band gap change is not responsible for the excellent photocatalytic activity of 3D-BiOCl under visible and natural white LED light. It is noteworthy that, unlike pristine BiOCl, 3D-BiOCl exhibits continuous absorption throughout the entire light spectrum, which may stem from the oxygen-vacancy-induced defect states in the forbidden band. As shown in the inset of Figure 5a, such a feature in the energy band results in a significant color shift in the material, intensifying the visible-light-induced photocatalytic activity of 3D-BiOCl [54]. Following the analysis of UV/Vis absorbance, the charge transfer and recombination behaviors were assessed using electrochemical impedance spectra (EIS) and PL emission spectra, respectively, as illustrated in Figure 5b,c. The EIS Nyquist diagrams of 3D-BiOCl, as revealed in Figure 5b, have a smaller curvature radius than BiOCl, showing that the defective hierarchical structure lowers the charge transfer resistance of 3D-BiOCl [55,56,57]. Furthermore, the fluorescence intensity of 3D-BiOCl in the 350–550 nm bands is substantially weakened compared with that of BiOCl, as seen in Figure 5c. In general, the PL intensity is dictated by the recombination rate of photo-generated carriers; a weak PL intensity suggests a low recombination rate, implying that the hierarchical structure of 3D-BiOCl facilitates the transit of photo-generated carriers, so as to effectively suppress photo-generated charge recombination [58,59,60]. It is generally believed that strong light absorption, high carrier transfer efficiency, and low carrier recombination would result in high photo-generated carrier concentrations in a sample [61,62]. To corroborate this argument, using a typical three-electrode cell, we have compared the transient photocurrent responses of the acquired samples over three on/off cycles. Both BiOCl and 3D-BiOCl exhibit a swift response when the light is switched on or off, as shown in Figure 5d, and do not display an apparent decrease in current density between cycles, showing continuous and sustainable production of the photo-generated carriers by samples. In addition, as expected, under the identical testing conditions, 3D-BiOCl is characterized by higher photocurrent intensities than pure BiOCl. This means that 3D-BiOCl can create more carriers during the photocatalytic process, thus making it a more active photocatalyst than BiOCl.

## 4. Conclusions

In this study, we developed a method for fabricating 3D hierarchical defective BiOCl at ambient pressure through etching-recrystallization with boiling EG as a treatment solution. The results indicate that the 3D-BiOCl photocatalyst not only possesses a hexagonal prism-like morphology at micron scale that arises from the assembly of BiOCl nanosheets, but also contains abundant oxygen vacancies, validated by SEM, XRD, Raman, and EPR analyses. In accordance with BET, UV/Vis absorbance, and photo-electro-chemical results, 3D-BiOCl was endowed with a higher specific surface area, better light harvesting capabilities, and more efficient separation and transfer of photo-excited charges compared with pristine BiOCl. As a result, 3D-BiOCl presents a remarkable photocatalytic activity for TC-HCl degradation in both visible light and natural white LED light. Furthermore, the target photocatalyst demonstrated an extraordinary recycling potential thanks to its hierarchical structure. In light of its simple and low-energy requirements preparation procedure, high efficiency in pollutants’ degradation, and ease of recycling properties, we believe 3D-BiOCl has a promising future in a variety of fields, including decoration materials and wastewater treatment.

## Figures and Tables

**Table 1:** Comparison of the degradation kinetic constants of TC for various photocatalysts.

Types of Photocatalyst	Light Source	Concentration of TC-HCl (mg/L)	Photocatalyst Dosage (g/L)	kinetic Constants (min^–1^)	References
BiOCl/Bi_2_Ti_2_O_7_	Simulated solar light	50	1	0.016	[42]
BiPO_4_ Nanorod/Graphene	UV light	20	0.5	0.034	[43]
N–TiO_2_/CNO_NV_	Visble light (λ > 420 nm)	30	0.4	0.017	[44]
AgBr–TiO_2_–Pa	Visble light (λ > 420 nm)	20	0.5	0.019	[45]
BiOCl@CeO_2_	Visble light (λ > 420 nm)	10	0.5	0.015	[46]
Se/BiOCl	Visble light (λ > 420 nm)	10	0.5	0.022	[47]
C/BiOCl	Visble light (λ > 420 nm)	10	0.5	0.014	[48]
BiOCl	Visble light (λ > 420 nm)	10	0.4	0.007	[49]
MWCNTs/Bi_4_O_5_I_2_	Visble light	20	0.2	0.012	[50]
3D–BiOCl	Visble light (λ > 420 nm)	30	1	0.025	This work

## Data Availability

The data presented in this study are available on request from the corresponding authors.

## Supplementary Materials

The following supporting information can be downloaded at: https://www.mdpi.com/article/10.3390/nano12152700/s1, Figure S1. UV–Vis spectrum of TC–HCl.; Figure S2. SEM picture of BiOCl.; Figure S3. SEM pictures of 3D–BiOCl.; Figure S4. Plots of (αhν)–1/2 versus photon energy (hν) for BiOCl (blue) and 3D-BiOCl (red).
