# Supplementary material for: Construction of Hexagonal Prism-like Defective BiOCL Hierarchitecture for Photocatalytic Degradation of Tetracycline Hydrochloride
Source: Nanomaterials (Basel). 2022 Aug 5;12(15):2700. doi: 10.3390/nano12152700 (PMC9370337; doi:10.3390/nano12152700)
Supplement: Supplementary file 1 [file nanomaterials-12-02700-s001.zip › nanomaterials-1840722-supplementary.pdf]

nanomaterials

MDPI

Supplementary Material

Construction of Hexagonal Prism-Like Defective BiOCL Hierarchitecture for Photocatalytic Degradation of Tetracycline Hydrochloride

Lijun Hu [1,2,*], Zhichao Ding [1,2], Fei Yan [1,2], Kuan Li [1,2], Li Feng [3], and Hongqing Wang [1,2,*]

1 School of Chemistry and Chemical Engineering, University of South China, Hengyang 421001, China
2 Hunan Key Laboratory for the Design and Application of Actinide Complexes, University of South China, Hengyang 421001, China
3 School of Civil and Transportation Engineering, Guangdong University of Technology, Guangzhou 510006, China
* Correspondence: lijun.hu@usc.edu.cn (L.H.); hqwang@usc.edu.cn (H.W.)

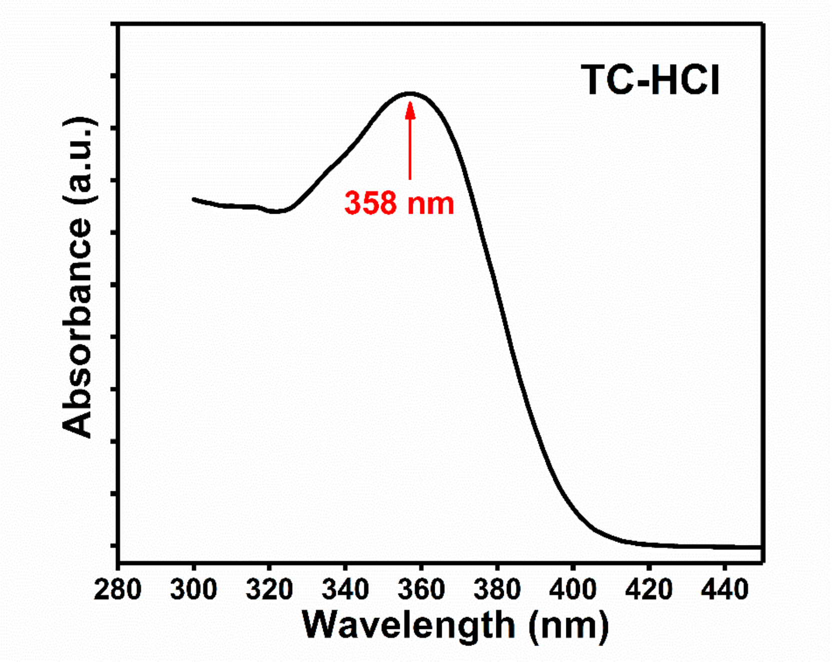

Figure S1. UV–Vis spectrum of TC–HCl.

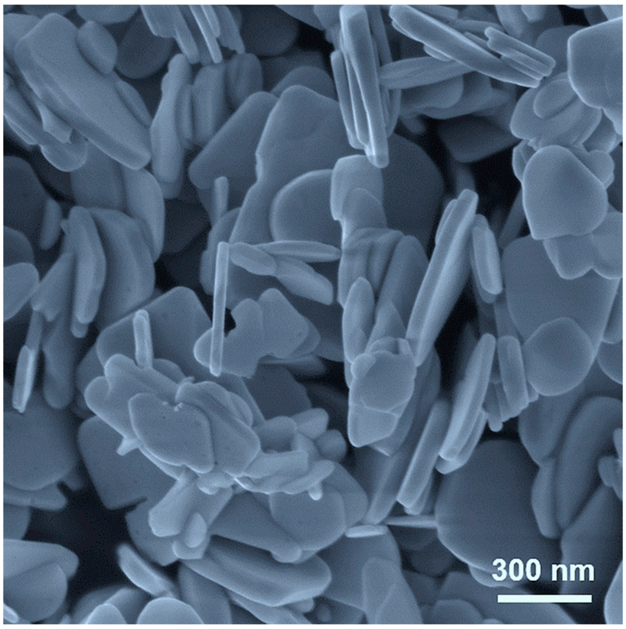

Figure S2. SEM picture of BiOCl.

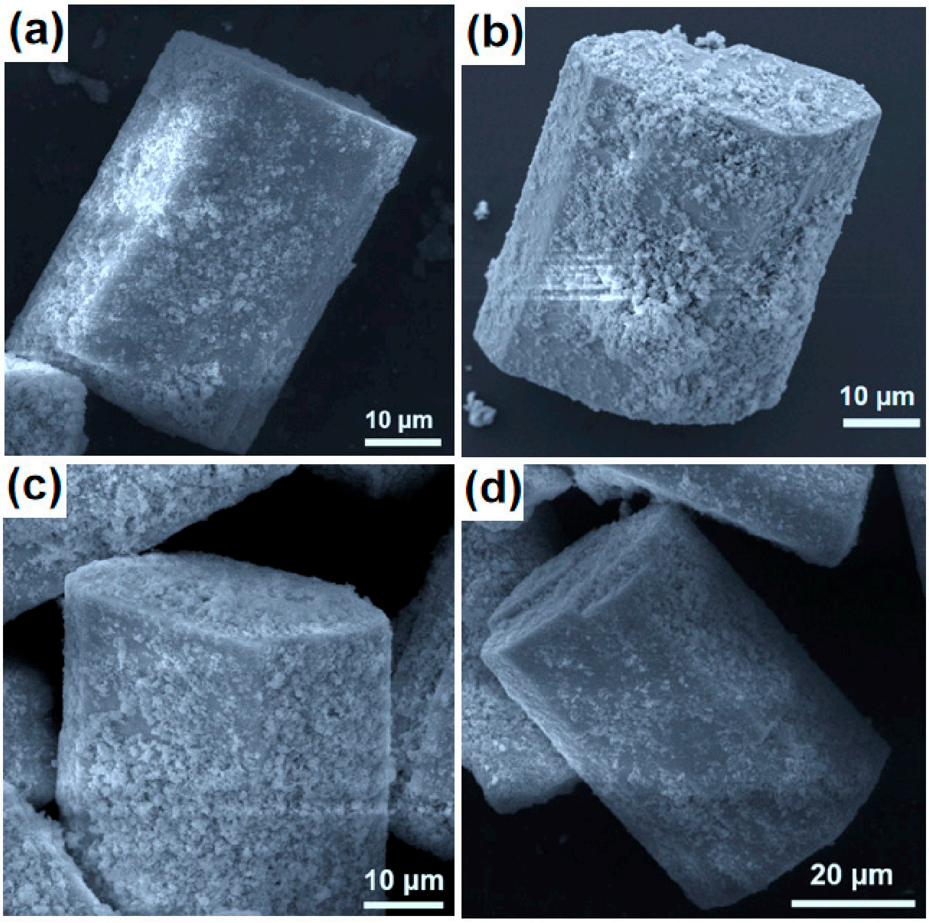

Figure S3. SEM pictures of 3D–BiOCl.

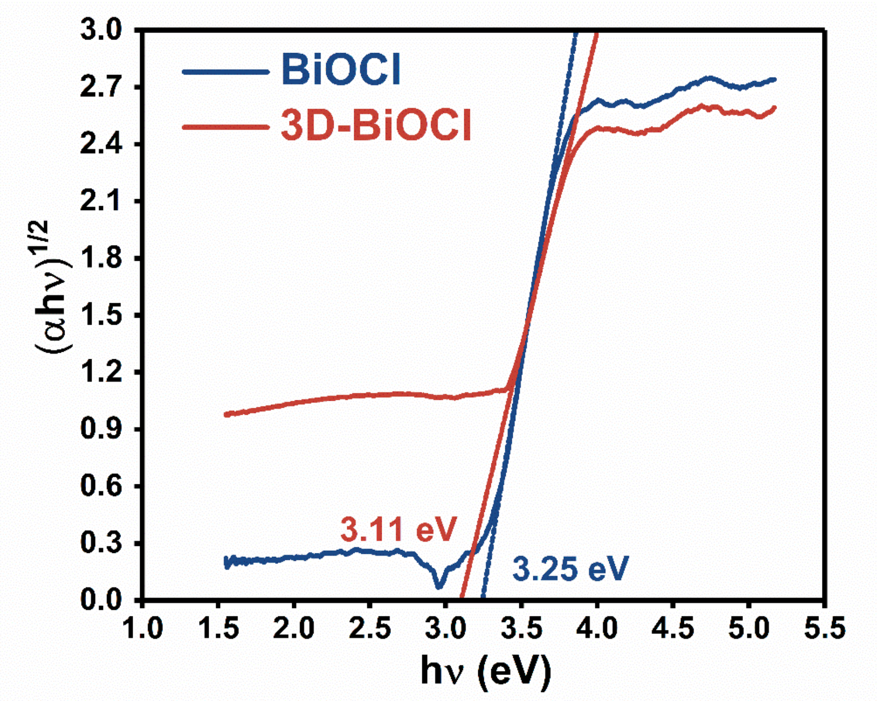

Figure S4. Plots of $(\alpha h\nu)^{-1/2}$ versus photon energy (hν) for BiOCl (blue) and 3D-BiOCl (red).
